# The Importance of Microhabitat for Biodiversity Sampling

**DOI:** 10.1371/journal.pone.0114015

**Published:** 2014-12-03

**Authors:** Zia Mehrabi, Eleanor M. Slade, Angel Solis, Darren J. Mann

**Affiliations:** 1 Biodiversity Institute, Department of Zoology, University of Oxford, Oxford, United Kingdom; 2 Hope Entomological Collections, Oxford University Museum of Natural History, Oxford, United Kingdom; 3 Wildlife Conservation Research Unit, Department of Zoology, University of Oxford, Abingdon, United Kingdom; 4 Spatial Foodweb Ecology Group, Department of Agricultural Sciences, University of Helsinki, Helsinki, Finland; 5 Unidad de Artrópodos, Instituto Nacional de Biodiversidad, Santo Domingo de Heredia, Costa Rica; Universidade de São paulo, Brazil

## Abstract

Responses to microhabitat are often neglected when ecologists sample animal indicator groups. Microhabitats may be particularly influential in non-passive biodiversity sampling methods, such as baited traps or light traps, and for certain taxonomic groups which respond to fine scale environmental variation, such as insects. Here we test the effects of microhabitat on measures of species diversity, guild structure and biomass of dung beetles, a widely used ecological indicator taxon. We demonstrate that choice of trap placement influences dung beetle functional guild structure and species diversity. We found that locally measured environmental variables were unable to fully explain trap-based differences in species diversity metrics or microhabitat specialism of functional guilds. To compare the effects of habitat degradation on biodiversity across multiple sites, sampling protocols must be standardized and scale-relevant. Our work highlights the importance of considering microhabitat scale responses of indicator taxa and designing robust sampling protocols which account for variation in microhabitats during trap placement. We suggest that this can be achieved either through standardization of microhabitat or through better efforts to record relevant environmental variables that can be incorporated into analyses to account for microhabitat effects. This is especially important when rapidly assessing the consequences of human activity on biodiversity loss and associated ecosystem function and services.

## Introduction

Understanding the spatiotemporal patterns in species distributions is critical for implementing conservation strategy [1]. However, demand for biodiversity data far exceeds the resources available for its collection [2], [3]. Furthermore, what can be inferred from the data that are available is wholly dependent on the scale at which they are collected and analyzed [4], [5]. This means that effective conservation prioritization directly depends on the fidelity of methodologies used in biodiversity assessments.

In order to efficiently and cost effectively map or monitor diversity the use of indicator groups is necessary [6]. Much research has shown that certain insect groups make good indicator taxa [1], [6], [7]. Dung beetles (Coleoptera: Scarabaeidae: Scarabaeinae) are one such group, and are widely recognized as an effective taxon that can be used in biodiversity assessments across the world [8], [9]. They are a key component of many tropical and temperate ecosystems, play important roles in ecosystem functioning (such as nutrient cycling, secondary seed dispersal, and parasite control), are amenable to rapid sampling, and are complemented by an active group of taxonomists [8], [10], [11]. Moreover, the response of dung beetles to environmental variables has been extensively documented [12]–[17], and they show marked responses to anthropogenic disturbance [9], [18], [19].

The standard methodology for trapping dung beetles uses dung baited pitfall traps along uniform linear transects [8], [20]. These traps are usually standardized in terms of bait used, bait size, spacing between traps, and length of time before collecting; and this allows ecologists to account for differences in the response traits of species in the community they sample. However the placement of the trap is rarely standardized. Accounting for species-specific responses to microhabitat is particularly important for non-passive sampling methods, such as baited traps or light traps, used for many important indicator groups, such as dung beetles, butterflies and moths. Differences in species’ responses to microhabitat, may affect trap effectiveness. In such cases the abundance and species composition of the sample will not relate to the abundance and species composition of the local community, but instead to a subset of the community that responds to the specific microhabitat conditions around the trap. Knowledge on the spatial resolution of non-passive traps is therefore essential to correctly relate samples to the surrounding habitat and landscape characteristics.

To the best of our knowledge, no studies to date have investigated the effects of trap placement and microhabitat preference of dung beetles on biodiversity metrics yielded from a typical field-sampling program. Here we specifically assess whether dung beetles show microhabitat level response traits, and if the current sampling methods used by ecologists to estimate dung beetle species diversity, biomass, and guild structure are robust to the effects of microhabitat variation. We analyze our results on the two different scales commonly used by ecologists: the trap and transect, in order to make our findings widely applicable. We discuss the implications of our findings for studies using dung beetles as an indicator taxon to assess habitat changes across multiple forest sites, and in particular those studies which link biodiversity to ecosystem functions and services.

## Methods

### Study site

**Figure 1:**
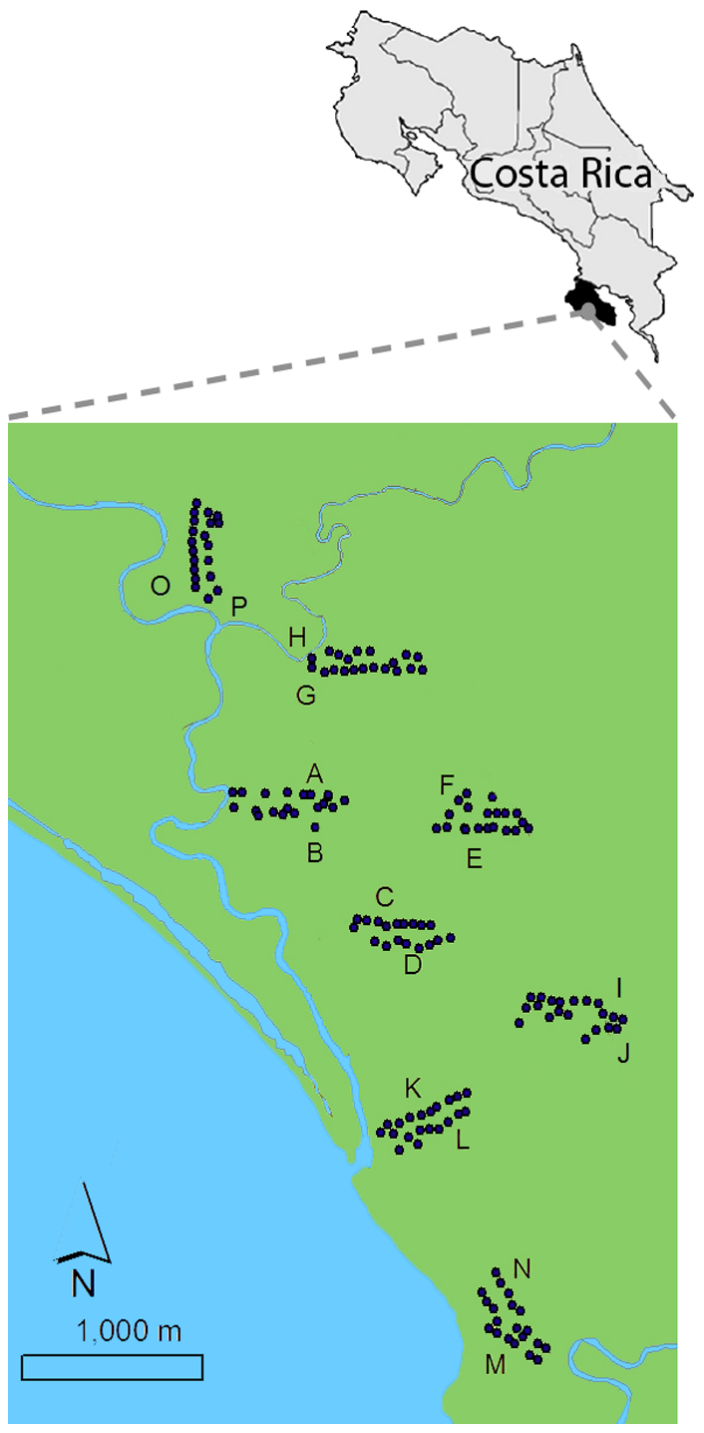
Study site. Sampling sites comprised of 8 pairs of transects (AB-OP). Each transect pair consisted of one transect with traps placed in a standardized microhabitat (“treatment” traps), and one transect with traps placed randomly at 50 m intervals as per methods usually employed in comparative studies of dung beetles (“control” traps) (see methods for further details). Dark blue dots represent sampling stations which are set locations along transects where single traps were serviced over a 72 hr period.

Fieldwork was carried out during July-August 2009, in the tropical wet forest surrounding Sirena Biological Station (8° 28′ 50″N 83° 35′ 20″W), Corcovado National Park, Osa Peninsula, Costa Rica (Fig. 1). Distinct seasons exist in the park with the vast majority of the annual rain falling during the months August-October, and least falling between months of January-March, totaling 5500 mm/year (INBIO unpubl. data). The mean average daily temperature is c. 25C. The area directly surrounding the station was subject to settlement during the 1940–50’s, with small pasture lands established during the 60s, and major clearings occurring between 1973–75, just prior to the parks establishment [58]. At the time of the study the sites used had been subject to 34 years of secondary regrowth in a matrix of contiguous primary old growth forest (Fig. S1). This study area supports diverse and abundant mammal populations [21], [22], which in turn provide a large resource base for dung beetle populations. In total 76 species of dung beetle have been recorded on the Osa Peninsula, 41% of the of the country’s 182 dung beetle species [23].

### Ethics

All necessary research and export permits were obtained for this project from Ministerio de Ambiente, Energía y Mares de Costa Rica. A reference collection of the insect material collected is curated at the Instituto Nacional de Biodiversidad, Costa Rica.

### Experimental design

The study followed a simple paired design with two types of trap placement: one standardized to microhabitat (“treatment”) and one randomly placed (“control”) (explained below). Control transects consisting of 10 sampling stations (50 m apart) were installed in eight spatially independent sites (AB-OP) (sites 500 m apart) (Fig. 1). The control transects used in this study represent the recommended standardised protocol for recording dung beetle distributions and abundances (ScarabNet: http://scarabnet.myspecies.info/) with each trap placed along a linear transect at 50 m intervals, regardless of the surrounding microhabitat features. Each control transect was coupled with a separate treatment transect 50 m apart: where trap placement was standardized to 1 m directly South from the base of a 60–80 cm diameter tree that was not situated next to tree falls or large open areas. The treatment trap placement was made to non-randomly select local microhabitat environmental variables around the trap, relative to the controls, whilst being close enough to be considered as sampling the same macro-habitat as the control. Global Positioning Systems (Garmin GPSMAP 60CSx) were used to mark out both control and treatment transects. Suitable sampling stations (trap locations set up for repeated trap servicing over a 72 hr period) for the treatment transects were located either directly on the linear transect or at short perpendicular deviations from it. In all cases minimum trap distances of 50 m were maintained throughout the study.

Dung baited pitfall traps, were installed at each sample station. A 7.5 cm diameter plastic cup was placed with the rim flush to the soil surface and 1/3 filled with water and a scentless detergent (to break the surface tension). Each trap was baited with 25 g of homogenized pig dung wrapped in biodegradable cheesecloth and tied to a stick suspended over the cup. Traps were covered by one large, or two crossed leaves to protect the trap from rainwater and direct manipulation of the dung by the beetles. Omnivore dung is effective bait for trapping in the neotropics [15], [24], [25], and pig dung in particular was suitable for our site because of seasonal peccary movements in the study area at the time of year we were trapping [22]. Each transect pair was run for 72hours, re-baiting and collecting specimens every 24hours. Specimens were stored in 75% ethanol until material was sorted and identified.

### Environmental variables

Temperature and relative humidity changes over the 72hour trapping periods, logged every 30 minutes, were recorded using EL-USB-2 data loggers (Lascar Electronics Ltd, UK), which were tied to tent pegs and placed 2 cm above the ground and 22 cm to the West of a trap, at equidistant points along the transects (4 on each transect). At each trap canopy openness was recorded using the Canopy Scope method: a simple, rapid and reliable assessment of forest understory light [26]. Diameter at breast height (DBH) of all stems >5 cm within a 5 m radius of each trap were measured, and used to estimate above ground biomass using regression equations in Chave *et* *al*. [27]. Ground cover was visually assessed in a 4 m^2^ quadrat surrounding each trap, and relative proportions of bare ground, leaves, twigs, large woody debris, and litter layer depth (mm) was recorded. Soil was sampled down to a depth of 10 cm, (representing the most frequently utilized zone tunneled by dung beetles [Mann unpubl. data]), and classified into 5 classes using textural inference: very coarse (sand, loamy sand), coarse (sandy loam), medium (loam, silt, silt loam), fine (sandy clay loam, silty clay loam, clay loam), very fine (clay, silty clay, sandy clay) (1–5 respectively). Each transect line was mapped onto the land use prior to 1976 map, and classified as old growth forest (undisturbed), undercut forest (with intact canopy but widespread understory clearing), abandoned farmland (cleared and used for crops between 1940–75), or old pastures and clearings (prolonged use of pastures and clear cut areas) [58] (Fig. S1).

### Analysis

Material was identified to species level using the INBio reference collection and papers listed in [28]. Data from each trap day at a given sample station were treated cumulatively and amalgamated. Any ambiguities in the integrity of a pitfall trap sample, as a result of flooding or interference with bait, led to that pitfall trap sample being omitted from analysis, along with the corresponding pitfall trap sample from the parallel paired transect. Equitability of trap spatial distribution between the control and treatment transects was confirmed (after elimination of one problematic sample station pair) using variance tests of x and y components of the standard deviation ellipses for each transect pair [29].

We calculated all biodiversity metrics at the trap and at the transect level. The transect level metrics were calculated on cumulated trap species abundances of the 10 sample stations on each transect. To control for differences in species richness resulting from unequal sample sizes [30], individual-based rarefaction curves were plotted for each sample station based on 1000 randomized iterations using the package PAST, and interpolated species richness extracted at the lowest number of individuals in any one sample [31]. To check whether treatment vs. control sampling stations generated differences in total species richness estimates across sampling sites in the study area, Chao1 estimates and their asymmetrical confidence intervals were computed using the package EstimateS (Version 8.2, R.K.Colwell, http://purl.oclc.org/estimates). Simpsons Effective Diversity Index (1/D) was computed in PAST [31]. As the full complement of different functional groups has been found to play a role in maximizing ecosystem functioning in dung beetle communities [32], guild structure were analyzed by calculating the relative proportions of endocoprids (dwellers), paracoprids (tunnelers) and telocoprids (rollers). *A. panamensis* and *T. pilosum,* were excluded from guild structure analyses, due to uncertainty in their feeding behavior (Table 1). As biomass has also been shown to be important for ecosystem functioning [33], [34], biomass was estimated by calculating the mean mass per species from dried specimens (either the mean of 20 individuals per species, or all collected during the study if less than 20) to 0.1 mg on an ABS 220-4 analytical balance (KERN Ltd Germany).

**Table 1:** Coprophagic Scarabaeinae species trapped separated by functional group.

Functional group and species	Abundance	Biomass (g)
Tunnelers		
* Anomiopus panamensis* (Paulian)^¶^	**1** (1^†^)	0.0014
* Canthidium ardens* Bates	**1** (1^†^)	0.0034
* Ateuchus aeneomicans* (Harold)	**2 (**2^*^)	0.0081
* Coprophanaeus pecki* Howden and Young	**2 (**2^*^)	0.4917
* Canthidium sp* (n/a)	**2** (2^*^)	0.0030
* Onthophagus marginicollis* Harold	**3** (2^†^, 1^*^)	0.0049
* Sulcophanaeus noctis* (Bates)	**4** (4^†^)	0.2661
* Canthidium aurifex* Bates	**5** (5^†^)	0.0312
* Canthidium haroldi* de Borre	**6 (**2^†^, 4^*^)	0.0162
* Trichillidium pilosum* (Robinson)^¶^	**13** (3^†^, 10^*^)	0.0018
* Uroxys boneti* Pereira & Halffter	**21** (19^†^, 2^*^)	0.0012
* Onthophagus coscineus* Bates	**23 (**13^†^, 10^*^)	0.0021
* Dichotomius satanas* (Harold)	**24 (**12^†^, 12^*^)	0.3034
* Canthidium centrale* Boucomont	**33** (11^†^, 22^*^)	0.0283
* Dichotomius amicitiae* Kohlmann & Solis	**59** (19^†^, 37^*^)	1.0621
* Onthophagus praecellens* Bates	**185** (94^†^, 91^*^)	0.0068
* Onthophagus coriaceoumbrosus* Kohlmann & Solis	**678 (**327^†^, 351^*^)	0.0083
* Copris incertus* Say	**1,314** (648^†^, 646^*^)	0.0533
* Onthophagus acuminatus* Harold	**9,783 (**5467^†^, 4316^*^)	0.0050
Sub total	***12,148*** ** ***(*** *6,634* ^†^, *5,511* ^*^ ***)***	***198.6227*** * (90.1503* ^†^, *105.2861* ^*^ *)*
Rollers		
* Deltochilum parile* Bates	**1** (1^‡^)	0.0568
* Canthon subhyalinus* Harold	**3** (1^†^, 2^*^)	0.0056
* Canthon mutabilis* Lucas	**4** (4^†^)	0.0115
* Deltochilum gibbosum* (Fabricius)	**4** (1^†^, 3^*^)	0.6787
* Canthon humboldti* Solis & Kohlmann	**4** (1^†^, 3^*^)	0.0033
* Megathoposoma candezei* (Harold)	**17** (3^†^, 14^*^)	0.2606
* Canthon moniliatus* Bates	**97** (53^†^, 44^*^)	0.0082
* Canthon aequinoctialis* Harold	**3,865** (1,661^†^, 2,204^*^)	0.0600
Sub total	***3,993*** * (1724* ^†^, *2272* ^*^ *)*	***232.7869*** * (98.4237* ^†^, *138.3632* ^*^ *)*
Dwellers		
* Eurysternus mexicanus* Harold	**17** (8^†^, 9^*^)	0.0337
* Eurysternus plebejus* Harold	**99** (39^†^, 60^*^)	0.0108
* Eurysternus foedus* Guérin-Méneville	**123** (39^†^, 84^*^)	0.0589
* Eurysternus hamaticollis* Balthasar	**1, 364** (578^†^, 786^*^)	0.0843
Sub total	***1,603*** * (664* ^†^, *939* ^*^ *)*	***123.872*** * (51.7133* ^†^, *72.1587* ^*^ *)*
Total	***17, 744*** * (9,022* ^†^, *8722* ^*^ *)*	***559.2816*** * (243.4736* ^†^, *315.808* ^*^ *)*

Abundance is the total number of individuals trapped at 150 dung-baited pitfall traps over 332 trap days. ^†^ = control (non-microhabitat standardised traps;, * = treatment (microhabitat standardised traps-see methods for description). ^¶^ = uncertainty in feeding behavior classification given. Biomass is either the mean of 20 individuals/species, or all individuals collected during the study if less than 20.

Trap and transect biodiversity data were analyzed using linear mixed models, with microhabitat treatment as a fixed effect and site as a random effect, using the *lme4* package in R 3.0.2 [59]–[60]. Separate models were fit for rarefied species richness, Simpson’s effective diversity, biomass, abundance and guild structure (%rollers, %dwellers, %tunnelers). The importance of microhabitat for predicting differences in biodiversity responses was tested by comparing the fit of these models to null models with the microhabitat (treatment vs. control) term removed using likelihood ratio tests. Dweller, tunneller and roller proportions were logit transformed after [57], biomass and abundance square root transformed, and 1/D log transformed, to meet linear modeling assumptions.

Although this study was not designed to test for interactive effects between historic land use and microhabitat placement on dung beetle biodiversity, it is possible that the different successional trajectories of land use over 34 years on our site [58], influenced biodiversity responses. We tested for potential confounding effects of land use on microhabitat treatments in two ways. Firstly, we re-built the linear mixed effects models outlined above with an additional term including land use history (as an interactive random effect with site). We then compared models containing land use history information to nested models without this term, using likelihood ratio tests and inspection of Akaike information criterion values. We found that including land use history did not significantly improve our model fits, suggesting we should drop it from our analyses. Secondly, we trimmed our dataset so that land use history classes were equally represented by trap pairs along each transect, and re-run our analyses. The results from this trimmed dataset did not alter the main conclusions of the paper. Therefore, we present in this paper results on the full dataset without land use history effect terms explicitly noted in our models. A full dataset for the paper is deposited on Dryad digital repository [61].

In order to test which environmental variables differed between our microhabitat treatments we compared mixed models for each measured environmental response, with and without microhabitat (treatment vs. control) as a fixed effect (retaining site as a random effect). In order to test the differences between ordinal environmental responses (e.g. soil texture, canopy openness) between treatments vs. control we performed our likelihood ratio tests on cumulative link mixed models using the *ordinal* package in R [62]. The remaining response variables were analyzed using linear mixed models, some of which were transformation to meet linear modeling assumptions: ground cover proportions and relative humidity were logit transformed after [57], above ground woody biomass was square root transformed, and litter layer depth was log transformed. The extent to which the environmental variables that were found to differ between treatment and controls could account for microhabitat treatment effects on biodiversity responses was tested using model selection. Here, environmental variables were fitted as fixed factors and compared to models with and without microhabitat treatment terms. Temperature values were averaged across each transect for this latter analysis.

In order to compare differences in effect sizes between trap and treatment scales of analyses, and for the effect of microhabitat trap placement with environmental variation taken into account, we estimated effect sizes (Cohens *d*) and approximate 95% confidence intervals for microhabitat treatment effects using equation 22 from Nakagawa and Cuthill [35], where *d* = 0.2, *d* = 0.5 and *d* = 0.8 are rough guides to small, medium and large effects respectively [36]. Denominator degrees of freedom for these calculations were approximated (Satterthwaite’s) using *lmerTest* package [63] in R.

Differences in community composition across treatments and sites were investigated with a two-way Analysis of Similarity (ANOSIM) using Bray-Curtis distances in PAST [31] The analyses produce an “R” statistic for each level (ranging between 1 and 1, where 1 indicates distances between groups are far greater than those within groups), which was evaluated for significance with 9, 999 permutations of group membership at the P<0.05 level. Differences in abundances of the species which contributed the most to community dissimilarity were tested using Wilcoxon Signed Rank tests, and *r* effect sizes calculated after Cohen [36] with *r* values *r* = 0.1, *r* = 0.3 and *r* = 0.5, equal to small, medium and large effects respectively.

## Results

### Community composition

17,744 specimens, representing 332 trap days were included in the analysis. A total of 31 species, with four species specific to control traps and five specific to treatment traps (Table 1). Community composition differed to a very small degree between control and treatment traps (R stat = 0.07, P = 0.006), and a small degree between sites (R stat = 0.26, P<0.0001). The largest contributors towards dissimilarity between the microhabitat treatments where *O. acuminatus*, *C. aequinoctialis, E. hamaticollis* and *C. incertus*, which accounted for 48%, 23%, 9% and 8% of the total dissimilarity between treatments, respectively. Of these, we found obvious differences in abundance between the treatments for *C. aequinoctialis* (Wilcoxon signed rank test: W = 851, Z = −2.3387, P = 0.02, *r* = −0.27) and *O. acuminatus*, (Wilcoxon signed rank test: W = 1843, Z = 2.462, p = 0.01, *r* = 0.28), but not for *E. hamaticollis* (Wilcoxon signed rank test: W = 859, Z = −1.5897, P = 0.11, *r* = −0.18) or *C. incertus* (Wilcoxon signed rank test: W = 1145, Z = 0.007, P = 0.97, *r* = 8×10^−4^).

### Species diversity

**Figure 2:**
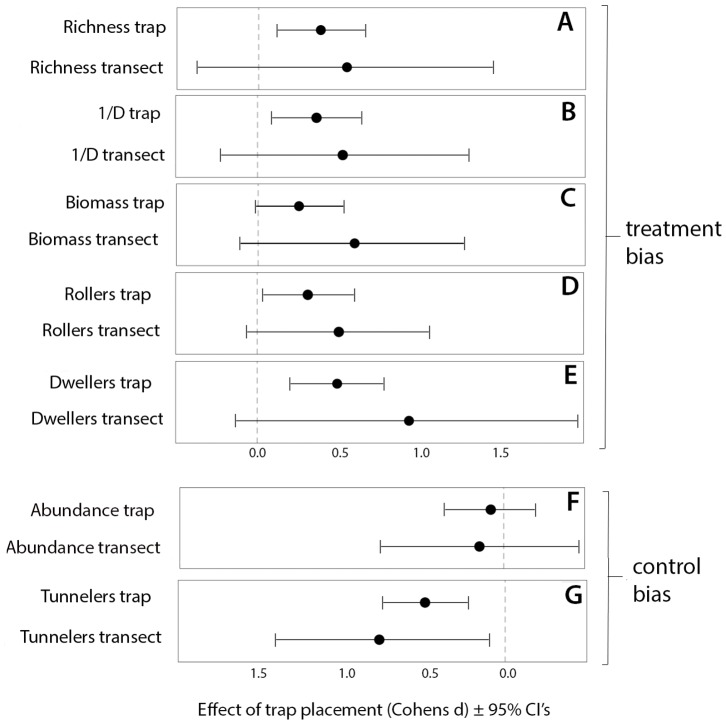
Effect of trap microhabitat on dung beetle biotic responses. The magnitude of the effect of trap placement (treatment = microhabitat standardized vs. control = non-standardized) on various biotic responses, where 0.2, 0.5 and 0.8 represent small, medium and large effects, respectively. Transect level metrics were calculated on cumulated trap species abundances of the 10 sample stations on each transect. Trap level metrics are calculated from cumulative species abundances collected over 72hours for each sample station (see Methods for details). Effect sizes are calculated from *t-values* generated in a linear mixed models framework with biotic variables as responses, site as a random effect, microhabitat treatment as a fixed effect. Total traps days = 332, Total number of individuals = 17,744.

**Figure 3:**
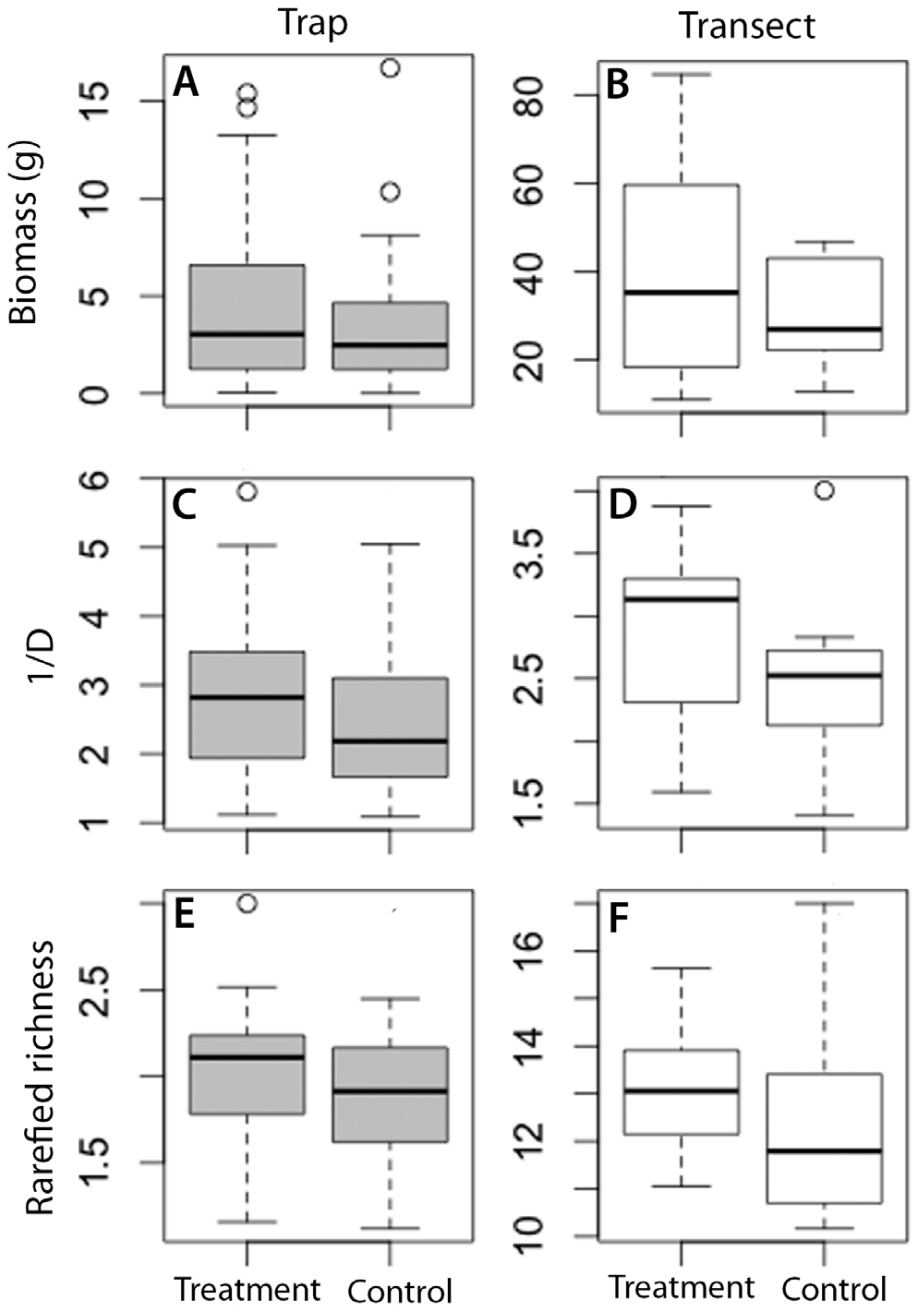
Distributions of dung beetle biotic responses to microhabitat treatments. Treatment = microhabitat standardized traps, control = non-standardized traps. Transect level metrics were calculated on cumulated trap species abundances of the 10 sample stations on each transect. Trap level metrics are calculated from cumulative species abundances over 72hours for each sample station (see Methods for details). The box represents the interquartile range, the line is the median, upper whisker is the 75th percentile and lower whisker the 25th percentile. All graphs are drawn from untransformed data. Total traps days = 332, Total number of individuals = 17,744.

Effective diversity (1/D) was higher in the treatment vs. control at the trap level (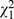 = 7.29, P = 0.006, *d* = 0.36). The effect of treatment on 1/D was more variable at the transect level (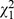 = 3.26, P = 0.07, *d* = 0.65) (Figs. 2B, 3B). Rarefied species richness was higher in treatment vs. control (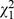 = 8.01, P = 0.005, *d* = 0.38). However, this was lost at the transect level (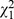 = 1.61, P = 0.2, *d* = 0.59) (Fig. 2A, 3E–F). Inspection of a plot of the confidence intervals of treatment and control Chao1 estimates, support this finding, showing a considerable overlap, indicating that the trapping methodologies did not yield large differences in minimum total species richness across the study area when controlling for abundance differences at each sample station (Fig. S2). Abundance did not differ between treatment and control at the trap (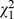 = 0.33, P = 0.56, *d* = −0.08), or the transect level (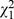 = 0.27, P = 0.59, *d* = −0.016) (Fig. 2F, Table 1).

### Functional metrics

**Figure 4:**
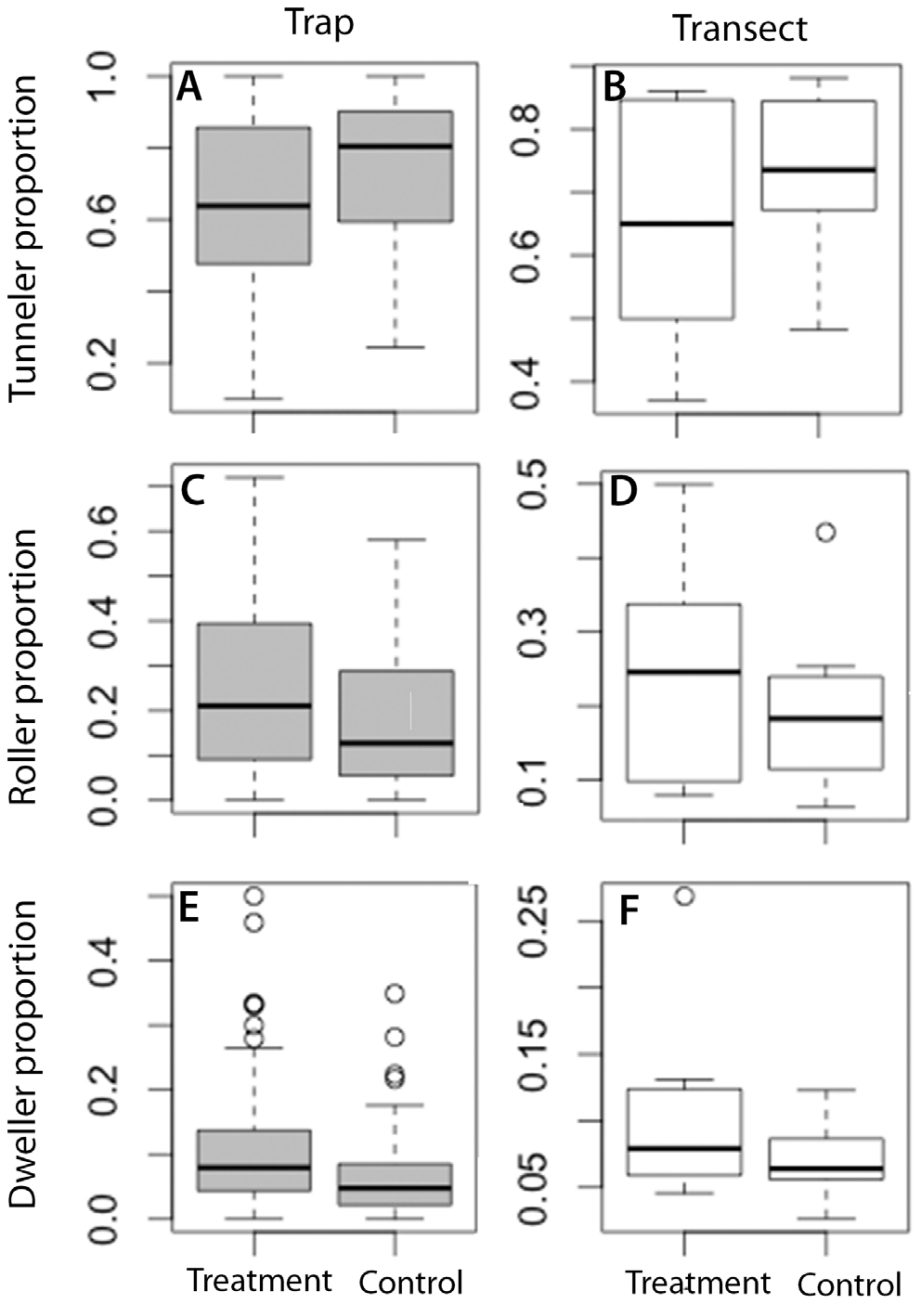
Distributions of dung beetle guild responses to microhabitat treatments. Treatment = microhabitat standardized traps, control = non-standardized traps. Transect level metrics were calculated on cumulated trap species abundances of the 10 sample stations on each transect. Trap level metrics are calculated from cumulative species abundances over 72hours for each sample station (see Methods for details). The box represents the interquartile range, the line is the median, upper whisker is the 75th percentile and lower whisker the 25th percentile. All graphs are drawn from untransformed data. Total traps days = 332, Total number of individuals = 17,744.

Biomass differences between treatments were not highly conclusive at the trap (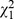 = 3.57, P = 0.058, *d* = 0.25), or the transect level (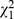 = 2.2, P = 0.13, *d* = 0.42) (Figs. 2C, 3C). There was however a clear difference in guild structure between treatment and control. Controls yielded a greater proportion of tunnelers relative to treatment, at the trap (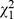 = 16.28 P<0.0001, *d* = −0.50) and the transect level (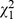 = 6.47, P = 0.012, *d* = −0.77) (Figs. 2G, 4A). Contrastingly, roller proportions were greatest in treatment trap vs. controls at the trap level (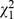 = 7.01 P = 0.008, *d* = 0.36), but effects of trap placement on rollers were less conclusive at the transect level (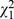 = 3.58, P = 0.058, *d* = 0.50) (Figs. 2D, 4B). Dweller proportions were also highest at treatment vs. control at trap (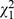 = 12.63, P = 0.003, *d* = 0.51), but as with rollers this effect was less conclusive at the transect level (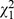 = 3.57, P = 0.059, *d* = 0.93) (Figs. 2E, 4C).

### Environmental variables

Treatment traps had 1385 kg (±408 SE) more above ground woody biomass (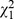 = 10.91, P<0.0001) relative to controls. Marginal differences were also found for temperature, with treatment traps 0.12**°**C (±0.06 SE) higher relative to controls (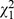 = 3.41, P = 0.064). No statistically clear differences were found between treatment and controls for bare ground cover (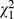 = 1.29, P = 0.25), large woody debris (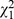 = 1.66, P = 0.19), leaf cover (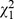 = 0.15, P = 0.69), twig cover (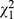 = 0.08, P = 0.77), litter layer depth (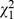 = 0.0009, P = 0.97), humidity (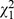 = 2.26, P = 0.13), canopy openness (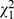 = 1.62, P = 0.20), or soil texture (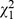 = 0.49, P = 0.48).

Despite the differences in above ground biomass and temperature between treatments, these environmental variables were unable to fully account for the effect of trap placement on 1/D (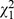 = 4.09, P = 0.027), species richness (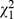 = 6.25, P = 0.012), the proportion of tunnelers (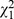 = 12.48, P = 0.0004), rollers (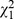 = 8.09, P = 0.004), or dwellers (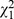 = 8.59, P = 0.003) (Fig. S3).

## Discussion

### Overview

Taxon-specific behavioral responses to spatial heterogeneity influence the way that biodiversity is studied and managed [4], [5], [37]. If the habitat is not defined from the perspective of the organism, then determining environmental parameters underlying population distributions can be problematic [5], [38], [39]. Here, we report the first study testing the effects of trap placement and microhabitat preference of dung beetles on commonly used metrics of biodiversity. We show that differences in biodiversity metrics can result from microhabitat trap placement over small spatial scales. This may have important implications for designing methodologies used for monitoring biodiversity and for studies investigating biodiversity-ecosystem functioning relationships using dung beetles as indicator taxa.

Firstly, if insect indicator taxa are used without representative sampling of the microhabitats they occupy, we may fail to compare like with like, which could in turn result in biases and lead to erroneous biodiversity valuations, or misguided conservation efforts. Secondly, in order to understand the functional roles underlying ecosystem processes performed by indicator groups, there is a need to account for the possibility that micro-scale variation in functioning could result from different microhabitat preferences of distinct functional guilds. In order to overcome these problems we suggest that sampling protocols for indicator taxa should incorporate standardization of microhabitat when placing traps, or record relevant environmental variables at the trap level, which can then be incorporated into analyses to account for microhabitat effects.

### Microhabitat preferences of dung beetles

The sensitivity of specific dung beetle species to irradiance, soil type, moisture, temperature, leaf litter, structural complexity, vegetative cover, and dung resource type are widely recognized [15], [40]–[43]. Despite the availability of resources, particular habitats will be avoided by particular taxa [44], [45]. Although we found that overall community composition was similar between treatments, some species (*O. acuminatus* and *C. aquinoctialis*) exhibited noticeable differences in the microhabitats they chose to visit. Moreover, we found three species (*Anomiopus panamenis, Canthon mutabilis* and *Canthidium ardens*) exclusively in non-standardized traps (although *A.panamensis* is a rare species that a rarely comes to pitfall traps at all).

The lack of treatment differences for most of the environmental variables we measured confirms our samples from traps along paired transects were obtained from much the same macrohabitat, and were even similar in terms of many commonly measured microhabitat environmental variables. The overall differences between species richness and diversity metrics between trap placements were small, inconsistent across scales of analysis, and probably biologically insignificant.

We found higher proportions of dweller and roller guilds, and lower proportions of tunnelers, at treatment versus control traps, indicating microhabitat preferences at the functional guild level. These differences were larger than those for species richness and diversity metrics, and, in the case of tunnelers, consistent over the scale of analysis. It may be expected that tunnelers, which directly bury beneath the soil, are be more likely to cope with open or warmer microhabitats than dwellers or rollers, which may be more prone to desiccation under these conditions [46], . Likewise, dwellers and rollers are better able to cope with soil obstruction than tunnelers [48], [46]. However, neither the humidity, nor ground cover parameters, which we measured, were significantly different between the standardized and non-standardized microhabitat trap placements in our study. Furthermore, the inability for temperature and above ground woody biomass to account for differences in guild structure, suggests that other factors, such as predation intensity, body size or sensory traits, may be more likely to influence the guild specific behavioral responses of dung beetles at this scale.

### Implications for monitoring ecosystem functioning

Guild structure, body size and biomass of dung beetles have been used to infer ecosystem functioning [42], [49], [50]. Studies have shown a lower effectiveness of small-bodied beetles for seed dispersal compared to large bodied individuals, and a disproportionate contribution of large bodied individuals for dung burial, even when high abundances of small-bodied individuals are present [19], [32]–[33], [52].

Despite the close proximity of the sample stations in the paired treatments, the differences in the proportions of functional guilds suggest that standardization of microhabitat will be important to link ecosystem processes to community functional composition. Ignoring microhabitat biases may hinder the development of a mechanistic understanding of the traits that underlie a particular function. For example, the majority of ‘larger’ species collected during the study (0.2–1.1 g dry weight) were found at microhabitat standardized traps, including the largest species *Dichotomius amicitiae*. This species belongs to the large nocturnal tunneler group, a group which makes significant contributions to community ecosystem functioning in both Asian and Neotropical rainforests [32], [34], [52]. In terms of biomass alone, *D. amicitiae* weighs 2.5 orders of magnitude more than the hyper-abundant *O. acuminatus* that largely accounted for the greater proportion of tunnelers found in non-standardized traps. The greater proportion of rollers found at standardized traps, especially large bodied species such as *Deltochilum gibbosum* and *Megathoposoma candezei*, are probably important for reducing clumping in seeds through dispersal away from the dung source, releasing plants from microsite limitation and negative density dependent pathogen attack [51], [53]. Thus, the conclusions drawn on the effects of particular functional guilds on ecosystem functions may be affected by the microhabitat in which sampling takes place.

### Implications for studies on habitat disturbance

The stenotopic nature of dung beetles and their rapid response to abiotic parameters, suggests local extirpation of many species as a result of alteration of microclimatic factors in heavily disturbed areas [44], [45], [54]–[55]. However, the consequences of anthropogenic disturbance on dung beetle species composition have been found to be variable, particularly at lower levels of forest disturbance [9], [19], [56]. One explanation why studies may differ in their findings on the effects of disturbance on communities is that comparative work does not consider microhabitat conditions when placing traps.

Our results show that although species diversity metrics were influenced by microhabitat treatments, the differences were small and unlikely to be important for studies on habitat disturbance. Contrastingly, the composition of functional groups differed with trap placement to a larger degree, and as changes in guild structure have been shown to have knock-on effects for dung removal and ecosystem functioning [32] we suggest that this difference is likely to be biologically significant.

We highlight that on a practical level, problems in biodiversity assessments using dung beetles are most likely to arise when trapping methodologies are employed under restricted resources, and are thus incapable of randomly sampling enough of the macrohabitat to guarantee that enough traps are placed to encompass the full range of microhabitats within. In such instances we suggest that standardizing trap placement, or recording microhabitat variables at the trap level and including these in analyses may help to mitigate these biases. However, it is clear from our study that we need a better understanding of exactly which environmental variables are driving fine scale responses. Caution also needs to be applied in making inferences from studies that do control for trap placement: as this will only sample a subset of the community of interest.

## Conclusions

In conclusion, we have shown that biases exist in current trapping protocols employed for the study of a widely used indicator taxon. We predict such biases to be most important in studies without sufficient replication and randomization along transects to properly represent the distribution of all potential microhabitats existing in a given macrohabitat. We found that the differences in species diversity responses to microhabitat conditions are subtle, but responses of functional guilds were more pronounced. We also found that the scale of analysis influenced the microhabitat bias. Thus, the impact of sampling methodology on decision-making may depend on whether functional or species richness based diversity measures are investigated, or at what scale they are analyzed. We suggest it may be possible to account for microhabitat preferences through standardizing trap placement or by including environmental parameters in analyses, but stress further knowledge of microhabitat preferences is needed to ensure relevant environmental parameters are measured in the field. A sharper focus on this topic would allow us to better understand the spatial patterns in animal ecosystem service provisioning.

## Supporting Information

Figure S1 **Study site including land use prior to 1976.**
(DOCX)

Figure S2 **Chao1 estimates for standardised (treatment) vs. non-standardised (control) microhabitat trap placement.**
(DOCX)

Figure S3 **Effect of trap microhabitat on dung beetle biotic responses with environmental variables considered.**
(DOCX)
