# Supplementary material for: The Importance of Microhabitat for Biodiversity Sampling
Source: PLoS One. 2014 Dec 3;9(12):e114015. doi: 10.1371/journal.pone.0114015 (PMC4254948; doi:10.1371/journal.pone.0114015)

**Figure S1. Study site including land use prior to 1976**. Sampling sites comprised of 8 pairs of transects (AB-OP). Each transect pair consisted of one transect with traps placed in a standardized microhabitat (“treatment” traps), and one transect with traps placed randomly at 50m intervals as per methods usually employed in comparative studies of dung beetles (“control” traps) (see methods for further details) (land use history map courtesy of Rob Plowes [56]).


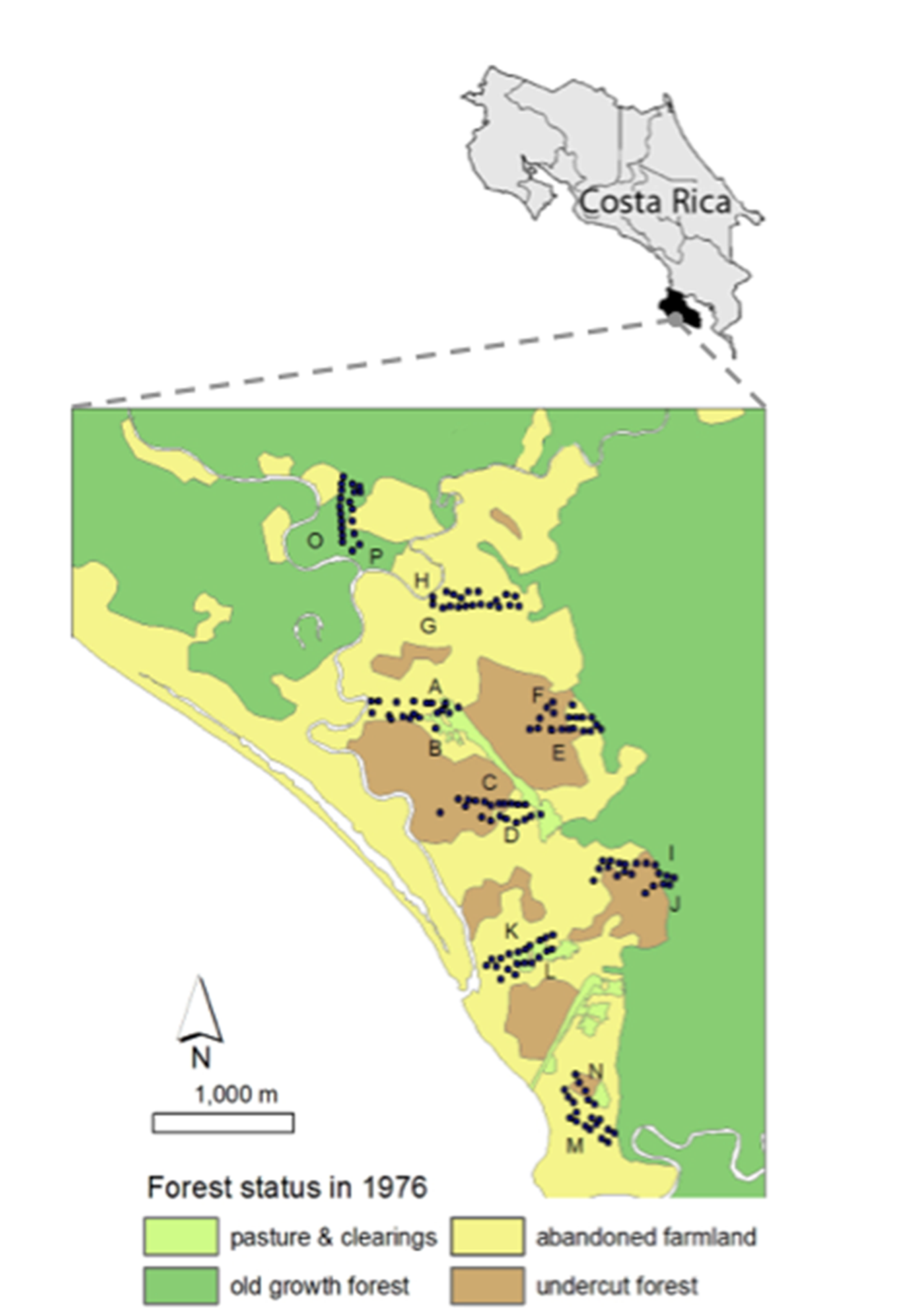

Supplement: Figure S1 — Study site including land use prior to 1976. (DOCX) [file pone.0114015.s001.docx]
