# Supplementary material for: The Importance of Microhabitat for Biodiversity Sampling
Source: PLoS One. 2014 Dec 3;9(12):e114015. doi: 10.1371/journal.pone.0114015 (PMC4254948; doi:10.1371/journal.pone.0114015)
Supplement: Figure S2 — Chao1 estimates for standardised (treatment) vs. non-standardised (control) microhabitat trap placement. (DOCX) [file pone.0114015.s002.docx]

**Figure S2** Chao1 estimates for standardised (treatment) vs. non-standardised (control) microhabitat trap placement. Overlap in treatment and control Chao1 estimates indicate that the trapping methodologies did not yield differences in minimum total species richness across the study area when controlling for abundance differences at each sample station. *
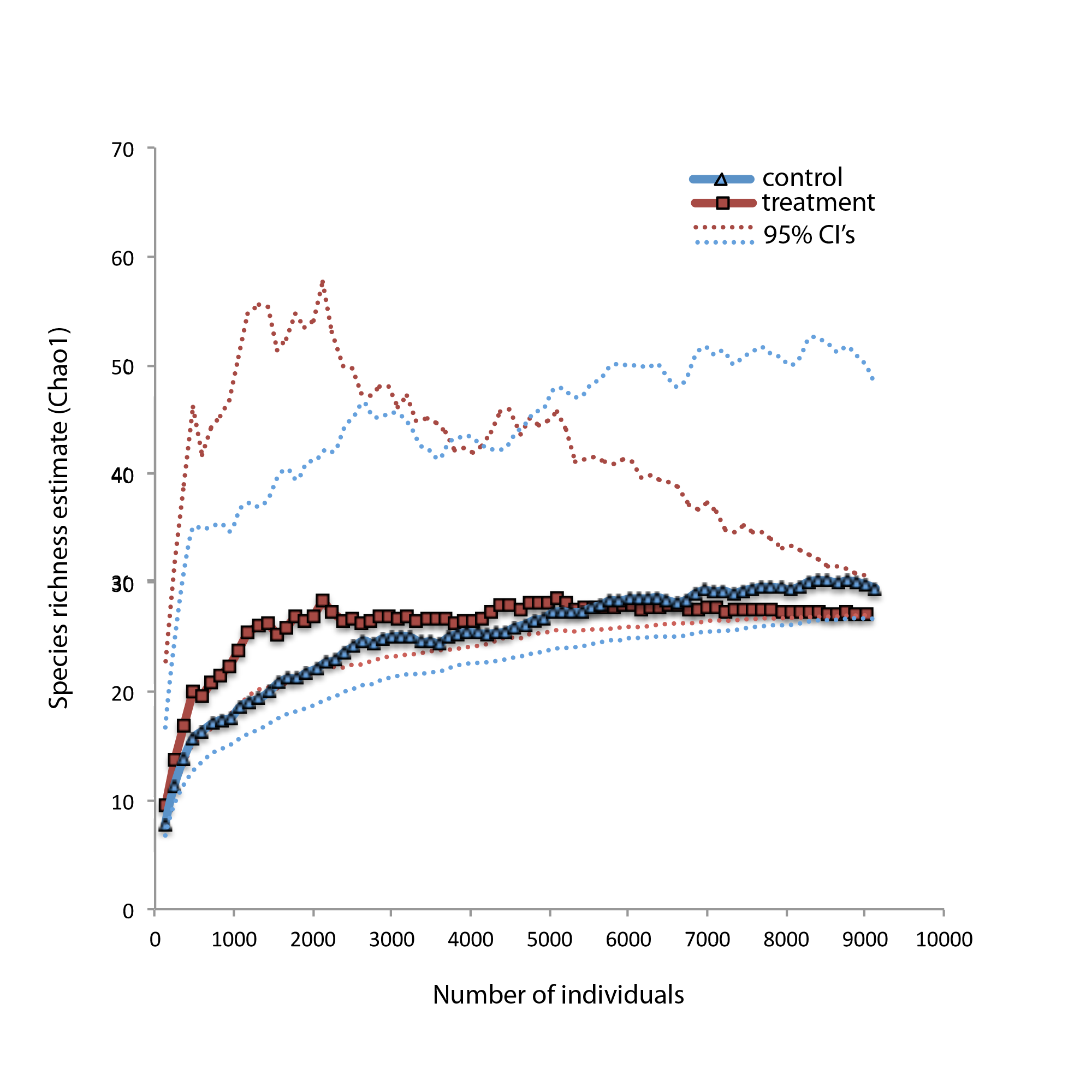
*
