# Supplementary material for: The Importance of Microhabitat for Biodiversity Sampling
Source: PLoS One. 2014 Dec 3;9(12):e114015. doi: 10.1371/journal.pone.0114015 (PMC4254948; doi:10.1371/journal.pone.0114015)

**Figure S3** Effect of trap microhabitat on dung beetle biotic responses**.** The magnitude of the effect of trap placement (treatment = microhabitat standardized vs. control = non-standardized) on various biotic responses, where 0.2, 0.5 and 0.8 represent small, medium and large effects, respectively. Effects for a given response ‘–e’ represent the magnitude of effect of trap placement *after* accounting for variation in trap measures of above ground woody biomass and temperature. Effect sizes are calculated from *t-values* generated in a linear mixed models framework with biotic variables as responses, site as a random effect, treatment as a fixed effect, and environmental variables as fixed covariates. All Total traps days= 332, Total number of individuals = 17,744.


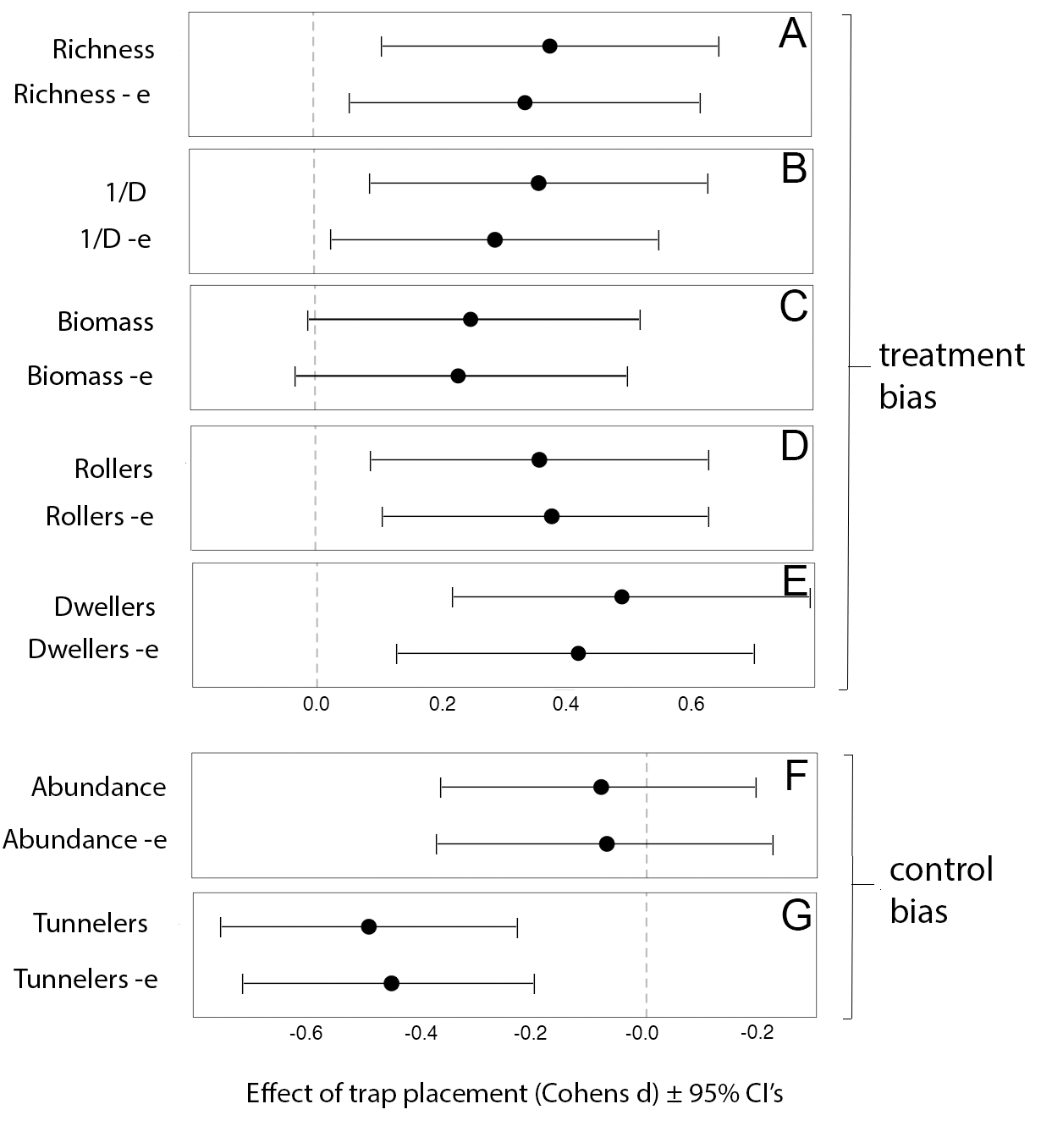

Supplement: Figure S3 — Effect of trap microhabitat on dung beetle biotic responses with environmental variables considered. (DOCX) [file pone.0114015.s003.docx]
